# Improving web-based respondent-driven sampling performance among men who have sex with men in the Netherlands

**DOI:** 10.1371/journal.pdig.0000192

**Published:** 2023-02-08

**Authors:** Sophie Diexer, Alexandra Teslya, Vincent Buskens, Amy Matser, Mart Stein, Mirjam E. Kretzschmar

**Affiliations:** 1 Julius Center for Health Sciences and Primary Care, University Medical Center Utrecht, Utrecht University, Utrecht, The Netherlands; 2 Department of Sociology, Utrecht University, Utrecht, The Netherlands; 3 Department of Infectious Diseases, Public Health Service (GGD) of Amsterdam, Amsterdam, The Netherlands; 4 Department of Internal Medicine, Amsterdam Institute for Infection and Immunity, Amsterdam UMC, Academic Medical Center, Amsterdam, The Netherlands; 5 National Coordination Centre for Communicable Disease Control, Centre for Infectious Disease Control, National Institute for Public Health and the Environment, Bilthoven, The Netherlands; The University of Sheffield, UNITED KINGDOM

## Abstract

Respondent-driven sampling (RDS) uses the social network of participants to sample people of populations that can be challenging to engage. While in this context RDS offers improvements on standard sampling methods, it does not always generate a sufficiently large sample. In this study we aimed to identify preferences of men who have sex with men (MSM) in the Netherlands regarding surveys and recruitment to studies with the subsequent goal of improving the performance of web-based RDS in MSM. A questionnaire about preferences with respect to various aspects of an web-based RDS study was circulated among participants of the Amsterdam Cohort Studies, a study among MSM. The duration of a survey and the type and amount of participation reward were explored. Participants were also asked about their preferences regarding invitation and recruitment methods. We used multi-level and rank-ordered logistic regression to analyze the data and identify the preferences. The majority of the 98 participants were older than 45 years (59.2%), were born in the Netherlands (84.7%), and had a university degree (77.6%). Participants did not have a preference regarding the type of participation reward, but they preferred to spend less time on a survey and to get a higher monetary reward. Sending a personal email was the preferred option to getting invited or inviting someone to a study, while using Facebook messenger was the least preferred option. There are differences between age groups: monetary rewards were less important to older participants (45+) and younger participants (18-34) more often preferred SMS/WhatsApp to recruit others. When designing a web-based RDS study for MSM, it is important to balance the duration of the survey and the monetary reward. If the study takes more of a participants time, it might be beneficial to provide a higher incentive. To optimize expected participation, the recruitment method should be selected based on the targeted population group.

## Author summary

In the Netherlands, men who have sex with men (MSM) are more likely to acquire HIV than the general population. However, due to stigmatization, some individuals of this population are hard to engage with standard sample methods. Respondent-driven sampling (RDS) can be used to recruit these people and subsequently obtain a representative sample of a population. RDS is a sampling method that uses social networks of participants to recruit others. Here, we used a questionnaire to find preferences of MSM regarding duration and incentives of surveys and recruitment methods to improve the performance of web-based RDS in this population. We found that participants prefer to take part in a study that is shorter and offers a higher monetary reward. Thus, it might be beneficial to provide a higher reward if a study takes more time. Furthermore, we found that a personal email was the preferred option to get invited or invite someone to a study. However, there are differences between age groups, with younger men having a higher preference for SMS/WhatsApp invitations than older men. Therefore, the recruitment method should be chosen depending on the specific subgroup of MSM targeted in a study.

## Introduction

In the Netherlands, the majority (63%) of new HIV diagnoses in 2021 were in men who have sex with men (MSM) [1]. Therefore, MSM continue to be a key population for HIV transmission prevention. Due to stigmatization it can be hard to engage different sub-populations of MSM with standard methods to assess the medical and sexual needs as well as up to date information about HIV prevention [2]. Respondent-driven sampling (RDS) is a sampling method that uses the social network of participants to obtain a representative sample of the whole MSM population [3, 4].

RDS starts with a convenience sample of the target population, so-called “seeds” who fill out a survey. Next these seeds are provided with a pre-specified number of coupons to recruit members of their social networks who are part of the population of interest. This is called the first “wave” of recruitment. Each participant is encouraged to invite their peers, who again invite peers, and so on, resulting in a series of waves [5, 6]. Coupons are used to track who recruits whom [5]. Incentives are used to encourage participation and successful recruitment of peers [3]. RDS combines a non-random sampling method with a statistical model to compensate for the sample not being collected randomly. This makes it possible to obtain estimates for the prevalence of traits of interest in the population. However, to obtain accurate estimates in the population under study, multiple recruitment waves need to be reached [3, 7]. Furthermore, with longer recruitment waves it can be possible to tap into populations that are less likely to be engaged by research, such as bisexual MSM and MSM that are not comfortable with their sexuality [8]. More recently, RDS has also been used to obtain information about contact networks by studying the correlations between recruiters and recruitees [9, 10]. The advantage of RDS in this context is that the collected data is not purely egocentric, but contains information provided by contact persons as well.

While RDS in MSM is used in many countries [4], in higher income countries RDS did not always perform well, resulting in poor recruitment rates [11–14]. This could be due to the fact that the incentives used to motivate participants were less effective in these countries [11]. Therefore the recruitment chains were short and the samples often remained small [11–13]. Higher monetary rewards have proven to be successful to create a sufficiently large sample in a study carried out in the United States [15], but this is very expensive and not feasible for many studies. High monetary rewards are also considered unethical and may lead to cheating behavior [16]. Another option to prevent recruitment chains from going extinct is to allow higher numbers of recruits per individual. Although this leads to a larger sample, it undermines the validity of the statistical framework of making estimates [9, 10, 17]. Other disadvantages of RDS that could limit recruitment are time and location related barriers.

Web-based RDS (WebRDS), an online variation of RDS, where recruitment is performed with digital tools, could possibly overcome some of the disadvantages of traditional RDS [16, 18]. Internet-based methods are easily accessible for participants in high income countries and are therefore an efficient possibility for researchers. However, WebRDS also has limitations, such as introducing selection bias due to different levels of internet access and frequency of use, e.g., between different age groups. Another limitation is the lack of interaction between researchers and participants, so there are less options to encourage peer-recruitment [18, 19]. In a study using web-based RDS among sexual minority women, the authors concluded that the lack of personal interaction between participants and researchers limited the participation rate in web-based RDS and resulted in very short recruitment chains (maximally two waves) [20]. In another study, online recruited seeds were more prevalent, but still recruited less participants than offline seeds [21]. Nevertheless, WebRDS has the potential to produce sufficiently large samples of MSM in high-income countries [17]. In a review on studies using WebRDS, Helms et al. found that sample sizes were between 19 and 3448 with recruitment chains of up to 29 waves [16].

We conducted a formative study to investigate preferences regarding specific attributes of a WebRDS study (duration of a survey, type and amount of participation reward), the preferred way of participants to get invited or recruit someone, as well as preferences regarding the amount of a recruitment reward. The goals were to understand the preferences for participating and for recruiting contacts of the target population, and subsequently improve the performance of future WebRDS studies in MSM in high income countries.

For eliciting preferences, we used discrete choice experiments (DCE), a technique to quantify individual preferences and a frequently used tool in health economics [22]. In DCEs, a participant must make choices between alternative options, which differ in their combination of factors under study. With many of these choices, preferences of participants with respect to the underlying factors, can be inferred. DCEs have the advantage that the influence of multiple attributes can be considered and that participants evaluate specific scenarios and are not asked about their preferences directly [23]. We propose that DCEs can also be used to identify preferences regarding studies and ways to improve them.

The results of the present study are being used in a larger project with the ultimate goal of conducting a web-based RDS study among MSM in the Netherlands to investigate contact patterns and Pre-Exposure Prophylaxis (PrEP) use in this population.

## Materials and methods

### Recruitment of participants

Participants were recruited from the Amsterdam Cohort Studies (ACS). The ACS was initiated in 1984 to investigate the HIV epidemic in MSM. At the time, our sub-study was conducted 2899 MSM had participated in the ACS throughout its existence, and approximately 760 MSM were in active follow-up, meaning they had at least one visit in the past two years. Every six months ACS participants were asked to fill in an extensive questionnaire and get tested for HIV and other sexually transmitted diseases [24].

A randomly selected subgroup (*N* = 300) of the cohort was invited via email to participate in our formative study and fill out a questionnaire. Not all ACS participants were invited to avoid survey fatigue. This study was completed in two rounds, the first one in summer 2019 and the second in late fall and winter of 2019. In each round, 150 participants were randomly picked from the cohort and invited to participate in our study. Participants invited in the first round were not invited again in the second round. The questionnaire was implemented through the GGD Amsterdam LimeSurvey platform, where participants could log into the survey using their ACS ID code. Reminders were sent after 14 days and after 25 days for the first round, and after 7 days for the second round. The ACS ID codes were used to link each survey to the corresponding ACS data. Participants were not incentivized for this study.

The following sociodemographic characteristics were assessed: age, country of birth, educational level, monthly income and place of residence. Age was binned into three categories: “18–34”, “35–44” and “45 and older”. Educational level was categorized as holder of a university degree, “Yes” or “No”. Income was indicated by the monthly income in one of five categories: “≤€950”, “€951-€1300”, “€1301-€1700”, “€1701-€2950” and “>€2950”. Place of residence was categorized with respect to Amsterdam, “Yes” or “No”, and country of birth was categorized as being born in the Netherlands, “Yes” or “No”.

### Questionnaire

**Fig 1:**
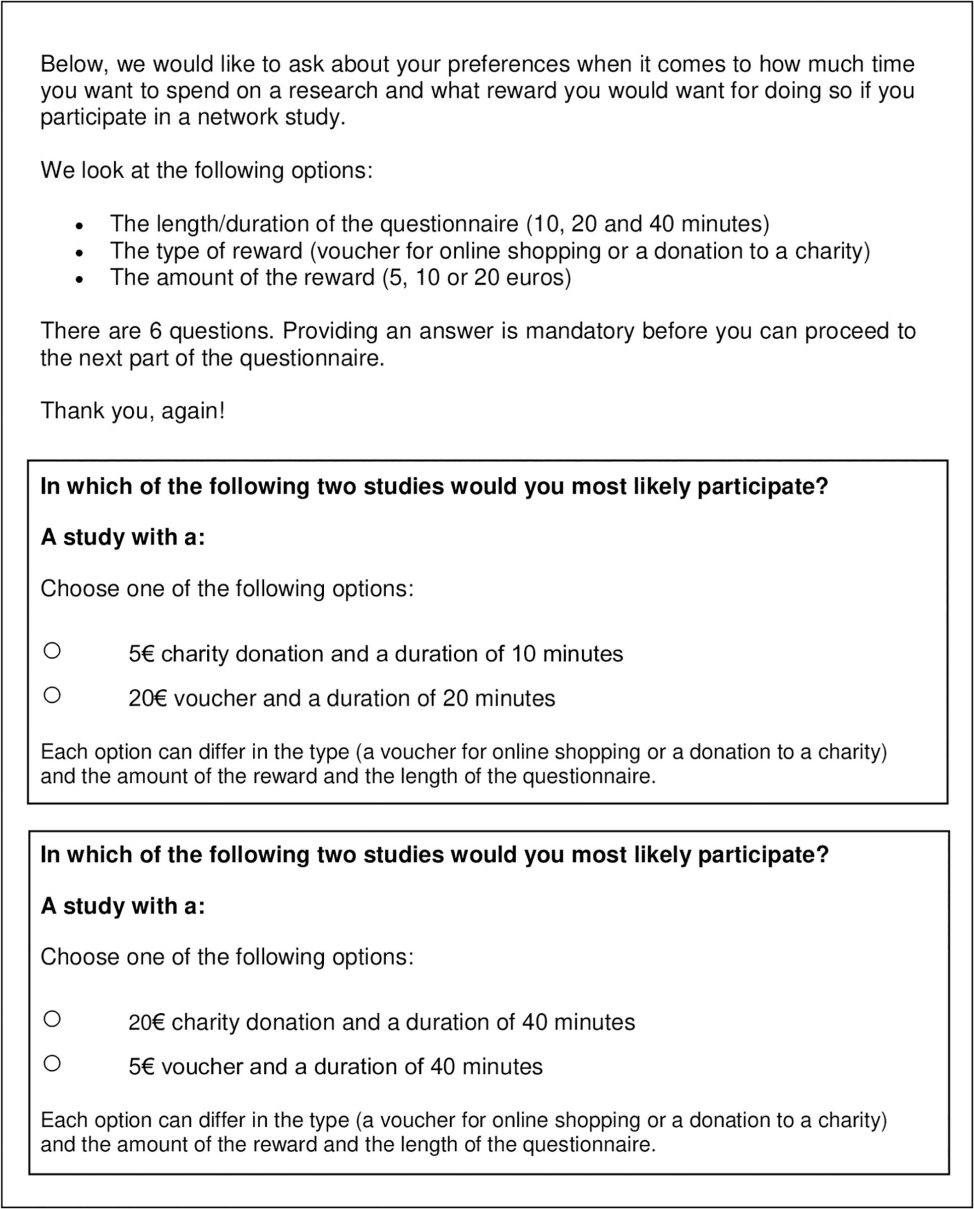
Example of discrete choice question as presented to participants.

The questionnaire was designed to investigate the preferences of participants regarding various aspects of an RDS study. These aspects included the actual study/questionnaire and forwarding process. The original questionnaire presented to the participants was in Dutch. The English translation of the questionnaire can be found in S1 Text. To study the preferences of participants with respect to the survey characteristics we looked at the following attributes: the time required to fill out a survey, type (donation/voucher) and the amount of incentive offered. Participants were asked to specify their preference about each of these attributes separately. Then, we investigated relative importance of each of these attributes by using DCE. Using this method, we can determine the preference on the combination of attributes and possibly their interdependencies, rather than only on each attribute separately. Participants were presented with choices between hypothetical options (vignette), which were based on the attributes described above [25, 26]. In our DCE, the attributes describing the duration of the study and the amount of the reward had three levels and the attribute describing the type of the reward had two levels. The options for the type of the reward were either a voucher for online shopping or a donation to a charity. Fig 1 shows an example of a discrete choice question as seen by a participant in our study. Participants were asked to select the study they prefer out of two choices. The levels of attributes differed between those choices. In total the participants were asked to fill out six questions in this format with 12 different scenarios (Table 1).

**Table 1:** Overview of all DCE choices.

	Option 1			Option 2		
	Duration	Type reward	Amount reward	Duration	Type reward	Amount reward
Choice 1	10 minutes	donation	5 €	20 minutes	voucher	20 €
Choice 2	40 minutes	donation	20 €	40 minutes	voucher	5 €
Choice 3	10 minutes	donation	5 €	40 minutes	voucher	10 €
Choice 4	40 minutes	donation	20 €	10 minutes	voucher	10 €
Choice 5	20 minutes	donation	10 €	10 minutes	voucher	5 €
Choice 6	20 minutes	donation	5 €	40 minutes	voucher	20 €

To assess the preferences regarding the recruitment of other individuals, participants were asked how they would like to be invited to a study and would like to invite someone to it. For each, they were asked to rank four options of being invited/recruiting someone according to their preference from most to least desirable. The options were “a personal invitation via email”, “an anonymous invitation from the researcher”, “a personal invitation via SMS or WhatsApp” and “a personal invitation via Facebook”. Additionally, participants were asked how important seven given factors are to them in deciding if they want to invite someone to participate in a study ((1) “absolutely not important” to (5) “very important”). The factors are “The questionnaire does not last too long”, “The questions are not too intrusive and personal”, “There is sufficient reward for completing the questionnaire”, “I think the study is important”, “Privacy of contact is protected”, “I receive a good reward for sending the invitation”, and “The questionnaire is easy and fun to complete”. Finally, to determine participants preference regarding the reward for recruiting someone to a study, participants could select if they rather get a €5 reward for every person (maximum 3 people) they successfully invite to a study, or whether they want €10 if at least one person is successfully invited.

### Data analysis

Descriptive analysis was used to describe the socio-demographic characteristics of the participants and their preferences regarding the specific features of a study. To analyze the DCE questions we used multi-level logistic regression analysis. In this design, the vignette and not the respondent is the unit of analysis, therefore we used a random intercept per participant. The results of this analysis only give information about relative preferences. This means it can be shown how much one level of an attribute is preferred over another, but not the absolute preference of that attribute. For instance, it can be determined that less time spent on a study is preferred but not how much time specifically. Furthermore, given the selection of vignettes administered to the participants it cannot be determined whether people prefer one type of reward over the other independently of the other choice characteristics. The preference for the type of reward can only be analyzed in interaction with preference for either the amount of the reward or survey duration. To determine the preferences for the invitation and recruiting method we used rank-ordered logistic regression. In addition, subgroup analysis was performed for every regression analysis. *p*-values less than 0.05 were considered as statistically significant. The analysis was carried out using R software (Version 3.6.1, R Foundation), package “lme4” [27], and STATA (Version 16.1 StataCorp, College Station, TX).

### Ethical approval and informed consent

The current study was approved by the Medical Ethics Committee of the Amsterdam University Medical Center of the University of Amsterdam, the Netherlands as part of the ACS (MEC-07/182). Written informed consent was obtained from all participants at enrollment. All methods in the study were carried out in accordance with relevant guidelines and regulations.

## Results

### Participant characteristics

Of 300 invited participants, 112 (37.3%) filled in at least one question. The number of participants who answered questions gradually declined with every question. As the DCE questions were the first ones in the questionnaire, we report only on individuals who completed at least all DCE questions (*n* = 98). For each analysis we report the number of participants used. Participants who did not complete the survey did not differ from participants who did. Furthermore, our participants did not differ from the average ACS participant [1].

The majority of the participants were older than 45 years (59.2%), born in the Netherlands (84.7%), and lived in Amsterdam (85.7%) (Table 2). Additionally, most individuals (77.6%) had obtained a university degree, however there was one participant who did not report their education level. We imputed this missing value with the population median (“Yes, I have a university degree”). Around 40% did not disclose their income, 22.3% indicated that they had an income between €1701-€2950 per month.

**Table 2:** Demographics of participants included in main analysis (*N* = 98).

	N (%)
**Age (years)**	
18–34	19 (19.4%)
35–44	21 (21.4%)
45+	58 (59.2%)
**Born in the Netherlands**	
Yes	83 (84.7%)
No	15 (15.3%)
**Education**	
No University Degree	22 (22.4%)
University Degree	76 (77.6%)
**Living in Amsterdam**	
Yes	84 (85.7%)
No	14 (14.3%)
**Income (per month)**	
≤€950	8 (8.2%
€951-€1300	5 (5.1%)
€1301-€1700	6 (6.1%)
€1701-€2950	22 (22.4%)
>€2950	16 (16.3%)
Unknown	41 (41.8%)

### Preferences for an RDS study

In total 94 participants answered the questions about the specific features of a study. 54 participants (57.4%) indicated that the preferred time of completing a study questionnaire is between 10 and 20 minutes (S1 Table). More participants preferred the voucher (56.4%) over the donation (43.6%), and the majority (48.9%) of participants indicated that the amount of the participation reward should be at least €10. However, almost 20% indicated that they would participate regardless of the reward.

Table 3 shows the results of the multi-level logistic regression analysis (*N* = 98). The data suggest that participants preferred to take part in a study that is shorter and offers a higher incentive (Table 3, Model A). No significant differences could be found between type of reward and the amount of participation reward, and type of reward and the duration of a study. This means that neither the amount of the participation reward nor the duration of the survey are more important for a donation than for a voucher. We, therefore, did not include the type of reward in further models. A significant interaction of the amount of the participation reward and age could be found (Model B, *p* = 0.004). The amount of the participation reward was less important for older participants (45+). The interaction between the duration of the study and age was marginally significant (*p* = 0.052), suggesting that younger people (18–34) wanted to spend less time on a study than the 45+ age category. People in the lowest income category were willing to spend more time on a study (Model C).

**Table 3:** Multi-level logistic regression analysis examining the preferences of participants regarding an RDS study (588 vignettes, 98 participants).

	Model A		Model B		Model C	
	Coefficient	S.E.	Coefficient	S.E.	Coefficient	S.E.
**Fixed part**						
Amount reward	0.124*	0.060	0.090***	0.015	0.114***	0.013
Duration	-0.064*	0.032	-0.047***	0.009	-0.020	0.016
Duration x Type reward (Ref. Voucher)						
Duration x Type reward (Donation)	0.007	0.030				
Amount reward x Type reward (Ref. Voucher)						
Amount reward x Type reward (Donation)	-0.014	0.067				
*Interaction Effects*						
Amount reward x Age (Ref. 45+)						
Amount reward x Age (18–34)			0.122**	0.041		
Amount reward x Age (35–44)			0.024	0.030		
Duration x Age (Ref. 45+)						
Duration x Age (18–34)			-0.039°	0.020		
Duration x Age (35–44)			-0.019	0.017		
Duration x Income (Ref. ≤€950)						
Duration x Income (€951-€1300)					-0.006	0.016
Duration x Income (€1301-€1700)					-0.059*	0.025
Duration x Income (€1701-€2950)					-0.047*	0.026
Duration x Income (>€2950)					-0.031	0.019
Duration x Income (Unknown)					-0.045*	0.018
**Random part**	Variance	S.D.	Variance	S.D.	Variance	S.D.
Respondent level	0.323	0.568	0.325	0.570	0.334	0.578

° *p* < 0.1;

* *p* < 0.05;

** *p* < 0.01;

*** *p* < 0.001

### Preferences for invitation and recruiting

Table 4 shows the estimated results of the rank ordered logistic regression. 85 participants completed the question regarding their preferred method of getting invited, and 83 participants reported on their preferences regarding the recruitment method. The first model determines the preferences for the method used to get invited to a study. Getting invited through Facebook was treated as a base alternative, meaning the coefficients of the alternatives represent the relative preference over Facebook as the invitation method. A personal email is the preferred invitation method over the three other options. The personal SMS/WhatsApp was ranked second, an anonymous email third, getting invited through Facebook was the least preferred option. However, it was shown in the subgroup analysis that older people (45+) had a preference for a personal email. In the younger groups this preference was not as strong. Participants from 18 to 34 years old still slightly preferred the personal email over an SMS/WhatsApp, while participants from 35 to 44 preferred the anonymous email, followed by getting an SMS/WhatsApp (S2 Table).

**Table 4:** Analysis of the preferred Invitation/Recruitment method (*N* = 83).

	Invitation Method		Recruiting Method	
Variables	Coefficient	S.E.	Coefficient	S.E.
Personal Email	1.394***	0.211	1.236***	0.219
Anonymous Email	0.609**	0.204	0.893***	0.212
SMS/WhatsApp	0.799***	0.200	1.111***	0.215
Facebook^a^	0	0	0	0

* *p* < 0.05;

** *p* < 0.01;

*** *p* < 0.001

^a^ Base alternative

The preferences for recruiting someone to a study were determined in the second model. For recruitment, participants do not have a distinct preference as long as the recruitment is not done via Facebook (base alternative). Overall, the personal email was the preferred option, followed by an SMS/WhatsApp and an anonymous email. However, participants in the oldest category (Category 3: 45+) preferred the personal email, while the youngest participants preferred sending an SMS or WhatsApp to recruit someone to a study.

Out of the 89 participants who answered the questions about the preferred reward for inviting someone, 60% preferred to get €5 for every person they successfully invite rather than getting €10 if they invite at least one person. Participants were asked how important seven different factors are in deciding to forward an invitation to an RDS study to a social contact. The most important factor to invite someone to a study seemed to be that participants think that the study is important. Another important factor was that the privacy of the contact person is protected. The amount of the reward for sending an invitation seemed to be the least important factor (Table 5).

**Table 5:** Mean scores (M) and standard deviation (SD) of importance of factors to forward an invitation to an RDS study to a social contact.

How important are these factors to you to forward the invitation to participate in the network study to your social contacts? N = 91	M (SD)
I think the study is important	4.50 (0.60)
Privacy of contact is protected	4.44 (0.73)
The questionnaire does not last too long	3.88 (0.73)
The questionnaire is easy and fun to complete	3.71 (0.82)
There is sufficient reward for completing the questionnaire	2.90 (1.11)
The questions are not too intrusive and personal	2.77 (1.04)
I receive a good reward for sending the invitation	2.55 (1.08)

The scores are on a 5-point Likert scale, with (1) for absolutely not important and (5) for very important.

## Discussion

Our findings show that participants prefer to spend less time on a study and to receive a higher monetary participation reward, and these preferences do not change with the type of reward to be earned. The amount of the participation reward seems to be less important to older people than younger people. Using Facebook for inviting someone or being invited to a study was the least preferred option out of the four given options, rather participants would like to send or receive a personal email. However, there are differences between the age groups, as younger people prefer SMS/WhatsApp, for recruiting others, over an email. The amount of the recruitment reward is a less important factor for participants to forward an invitation to a social contact. The protection of the privacy of the contact person and that the study is relevant for the community are more decisive factors.

To our knowledge, this is the first study that attempts to identify preferences of MSM regarding a WebRDS study in a high-income setting. This may provide useful insights in improving performance of WebRDS, specifically WebRDS in the population of MSM. There are also limitations to this study, the main one being the small sample size of only 98 men. However, since the individual person is not the unit of the analysis for the DCE, this is not an issue for the analysis of these questions. On the other hand, small sample size poses a problem for identifying the preferences of invitation and recruiting, because even less people provided answers to those questions. Furthermore, the ACS population might not be a representative sample for the entire MSM population in the Netherlands. ACS participants are predominately highly educated with a Dutch background and therefore their preferences might not reflect the preferences of the general MSM population. Furthermore, ACS participants already participate in a study without getting an incentive, therefore we might underestimate the importance of incentives in this study. Another limitation could be the age of the participants. The majority of the participants is over 45 years old. If the target group of a future study is younger people, these findings might not reflect their preferences. Finally, the results might be sensitive to the attributes and operationalizations of these attributes chosen, limitations that impact all DCEs. Furthermore, the actual behavior might differ from the intentions reported in this survey.

RDS is particularly well suited to situations when random sampling techniques are not feasible, for example if populations are geographically dispersed, or if they are difficult to engage. Furthermore, WebRDS offers an improved way to target these types of populations. Unfortunately, past (web-based) RDS studies often had short recruitment chains [9, 10, 17, 20, 28–32]. The studies might not have been attractive enough for participants, or participants might be hesitant to invite peers to a survey, which is why it is important to know the preferences of participants to improve recruitment.

Our findings show that participants want to spend less time on studies, therefore it is better to keep questionnaires short. Our findings are in line with a previous study which suggested that the response rate is higher if a questionnaire is shorter [33]. Additionally, the authors of the study found that strategies such as personalization or sending reminders were indicated to be helpful in improving response rates [33]. Several WebRDS studies have reported sending reminders [9, 28, 30–32, 34–37], yet two studies still reported a sample size of under 100 [31, 32]. We found that, the duration of time required to fill in the questionnaire was also an important factor for participants to decide if they want to forward an invitation for a study to a social contact. Nevertheless, it can be hard to find a compromise between survey duration and collecting all the data that is needed for a study. Also, a balance needs to be found between difficulty and/or sensitivity of a question and the researchers’ need to get precise information. An option to motivate participants to take art in a study could be to increase the amount of the participation incentive. A review of WebRDS surveys found that studies that did not offer at least one guaranteed material incentive reached lower percentages of successfully recruiting participants and fewer waves, indicating that the amount of participation reward or another incentive is a big motivator for participants [16]. However, our results show that getting a high reward is actually the least important factor for forwarding an invitation. Nevertheless, the results of the DCE show that participants prefer to get a higher monetary reward for participating in the study themselves. This suggests that a higher incentive might be a reason to participate in a study, but would not be the most important thing when recruiting a social contact. Furthermore, the same review also showed that online recruitment was faster in studies, in which study populations were composed of university students, and suggested that this might be due to the fact that this population is more digitally literate and has a more extensive online network [16, 18, 38]. This conclusion corresponds to our findings that different age groups prefer a different recruitment strategy, which should be considered when designing a WebRDS study.

DCEs have long been used to measure preferences in economics, transportation, and public health and have shown to be a robust method for predicting behaviors and preferences [39]. It is a relatively cheap and quick method of quantifying preferences and is therefore appealing for investigating preferences of participants regarding participation in studies.

The findings of this study could provide valuable input to optimize web-based RDS studies among MSM in high-income countries. Future studies can seek to build on these findings and incorporate the preferences of MSM regarding different aspects of RDS. Furthermore, the relationship between the time it takes to fill in a survey and incentive should be explored further to improve response rates in general. Future studies could also assess whether the COVID-19 pandemic has changed attitudes of MSM towards participating in online RDS surveys.

## Conclusion

We showed that individuals in different age groups have different preferences with respect to characteristics of surveys and modes of invitation and peer recruitment. Therefore, the study and the sampling method should be adjusted to optimize expected participation for specific subgroups of MSM to be targeted. These findings improve the understanding of the preferences of the target population and subsequently improve participation in WebRDS studies.

## Supporting information

S1 Text Full questionnaire. English Translation.
(PDF)

S1 Table Preferences regarding the DCE options. (PDF)

S2 Table Analysis of the preferred Invitation/Recruitment method by Age group. (PDF)
