# Supplementary material for: Improving web-based respondent-driven sampling performance among men who have sex with men in the Netherlands
Source: PLOS Digit Health. 2023 Feb 8;2(2):e0000192. doi: 10.1371/journal.pdig.0000192 (PMC9931300; doi:10.1371/journal.pdig.0000192)
Supplement: S1 Text — English Translation. (PDF) [file pdig.0000192.s001.pdf]

# Preferences when participating in network studies

**Use a laptop, tablet or desktop to complete this survey.**

For more information, click on the video:

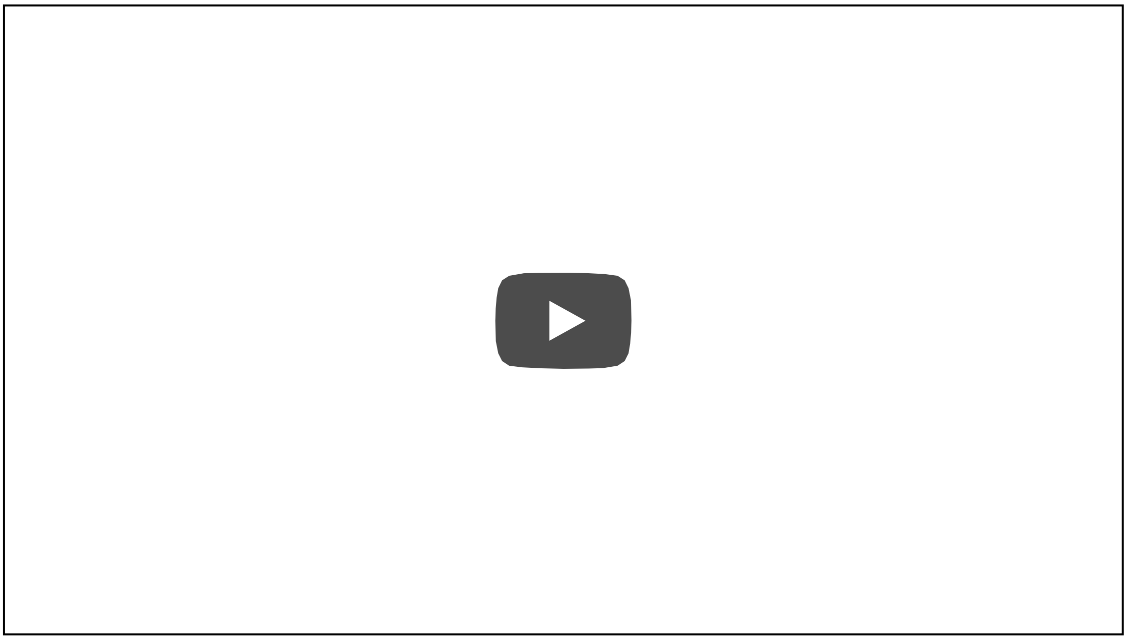

**This questionnaire is about the preferences of research participants such as yourself.**

We would like to ask you to participate in this short extra questionnaire about the preferences, willingness and motivation to participate in a special type of scientific research, a network study. The results of this research help us to develop this network study..

**What is a network study and why do we want to do it?** In a network study, people are asked to participate themselves and also to invite their social contacts. Network studies provide us with insight into the structure of the network, so that we can better understand how information such as prevention messages are distributed in a network. It also helps us understand how HIV and STIs can spread in a network.

**This survey.** This survey consists of filling in a short questionnaire in which we ask about your preferences when it comes to the time investment you want to make and the reward you would like to receive for participating in a network study.
This research is <u>not</u> a network study; we do not ask you to invite contacts. This questionnaire contains 30 questions and takes about 15 minutes to complete.

This research is part of the Amsterdam Cohort Studies and is carried out by the GGD Amsterdam and the UMC Utrecht.
Your data will be processed according to the Amsterdam Cohort Studies protocol and pseudonymized.

**Thank you for your participation!**

There are 29 questions in this questionnaire

# DCE

Below, we would like to ask about your preferences when it comes to how much time you want to spend on a survey and what reward you would want for doing so if you participate in a network study.

We look at the following options:

- The length / duration of the questionnaire (10, 20 and 40 minutes)
- The type of the reward (voucher for online shopping or a donation to a charity)
- The amount of the reward (5, 10 or 20 euros)

There are 6 questions. Providing an answer is mandatory before you can proceed to the next questionnaire section.

Thank you, again!

For more information, click on the video:

[]

**In which of the following two studies would you most likely participate? A study with a:**

*****

Choose one of the following options:

- ○ 5€ charity donation and a duration of 10 minutes
- ○ 20€ voucher and a duration of 20 minutes

Each option can differ in the type (a voucher for online shopping or a donation to charity) and the amount of the reward and the length of the questionnaire.

[]

**In which of the following two studies would you most likely participate?  A study with a:**

*

Choose one of the following options:

- 20€ charity donation and a duration of 40 minutes
- 5€ voucher and a duration of 40 minutes

Each option can differ in the type (a voucher for online shopping or a donation to charity) and the amount of the reward and the length of the questionnaire.

[]

**In which of the following two studies would you most likely participate?  A study with a:**

*

Choose one of the following options:

- 5€ charity donation and a duration of 10 minutes
- 10€ voucher and a duration of 40 minutes

Each option can differ in the type (a voucher for online shopping or a donation to charity) and the amount of the reward and the length of the questionnaire.

[]

**In which of the following two studies would you most likely participate?  A study with a:**

*

Choose one of the following options:

- 20€ charity donation and a duration of 40 minutes
- 10€ voucher and a duration of 10 minutes

Each option can differ in the type (a voucher for online shopping or a donation to charity) and the amount of the reward and the length of the questionnaire.

**[]**

**In which of the following two studies would you most likely participate?  A study with a:**

*****

Choose one of the following options:

- 10€ charity donation and a duration of 20 minutes
- 5€ voucher and a duration of 10 minutes

Each option can differ in the type (a voucher for online shopping or a donation to charity) and the amount of the reward and the length of the questionnaire.

**[]**

**In which of the following two studies would you most likely participate?  A study with a:**

*****

Choose one of the following options:

- 5€ charity donation and a duration of 20 minutes
- 20€ voucher and a duration of 40 minutes

Each option can differ in the type (a voucher for online shopping or a donation to charity) and the amount of the reward and the length of the questionnaire.

## AbsAcc

**[]**

**For the following situations, indicate how likely it is that you would participate in research.**

Choose the appropriate answer for each item:

| | certainly not | unlikely | perhaps | probably | certainly |
|---|---|---|---|---|---|
| 5€ charity donation, 40 minutes | ○ | ○ | ○ | ○ | ○ |
| 5€ voucher, 40 minutes | ○ | ○ | ○ | ○ | ○ |
| 20€ charity donation, 5 minutes | ○ | ○ | ○ | ○ | ○ |
| 20€ voucher, 5 minutes | ○ | ○ | ○ | ○ | ○ |
| 10€ charity donation, 20 minutes | ○ | ○ | ○ | ○ | ○ |
| 10€ voucher, 20 minutes | ○ | ○ | ○ | ○ | ○ |

**[]**

## How much time do you want to spend at most on completing a questionnaire?

Choose one of the following options:

- ○ Less than 10 minutes
- ○ 10 to 20 minutes
- ○ about 30 minutes
- ○ about 45 minutes
- ○ an hour or longer
- ○ the ideal maximum amount of time depends on something else.

Please explain your answer here:

## [] What would it depend on?

**Answer this question only if the following conditions are met:**
Answer was 'the ideal maximum amount of time depends on something else.' for question '8 [ideal time]' (How much time do you want to spend on completing a questionnaire?)

Select all options:

- ☐ Amount of the monetary stimulus
- ☐ Importance of the research to me
- ☐ Other: ______

[]

### What type of reward do you prefer?

Choose one of the following options:

- a donation to charity
- a voucher for online shopping

We use rewards to encourage participation in network sampling. Would you rather receive a reward such as a voucher to use online or a donation to a charity of your choice?

[]

### How high should the voucher be to motivate you to participate in a network study?

**Answer this question only if the following conditions are met:**
Answer was 'a voucher for online shopping' to question '10 [incentive]' (What type of reward do you prefer?)

Choose one of the following options:

- at least 5€
- at least 10€
- at least 20€
- more than 20€
- I participate regardless of the reward
- the minimum monetary reward would depend on something else

Please explain your answer here:

## []What would it depend on?

**Answer this question only if the following conditions are met:**
Answer was 'the minimum monetary reward would depend on something else' to question '11 [amountBon]' (How high should the voucher be to motivate you to participate in a network study?)

Choose one of the following options:

- how long the questionnaire is
- importance of the research to me
- Other

## []

## How high should the charity donation be to motivate you to participate in a network study?

**Answer this question only if the following conditions are met:**
Answer was 'a donation to charity' to question'10 [incentive]' (What type of reward do you prefer?)

Choose one of the following options:

- at least 5€
- at least 10€
- at least 20€
- more than 20€
- I participate regardless of the reward
- the minimum monetary reward would depend on something else

Please explain your answer here:

## []What would it depend on?

**Answer this question only if the following conditions are met**
Answer was 'the minimum monetary reward would depend on something else' to question '13 [amountDon]' (How high should the charity donation be to motivate you to participate in a network study?)

Only enter a comment if you choose an answer.

Select all possible answers and provide an explanation:

| | |
|---|---|
| ☐ how long the questionnaire is | |
| ☐ importance of the research to me | |
| ☐ otherwise, namely | |

## []What would motivate you to participate in a network study?

**Answer this question only if the following conditions are met:**
Answer was 'I will participate regardless of the reward' to question '11 [amountBon]' (How high should the voucher be to motivate you to participate in a network study?)

Enter your answer here:

## []What would motivate you to participate in a network study?

**Answer this question only if the following conditions are met:**
Answer was 'I participate regardless of the reward' to question '13 [amountDon]' (How high should the charity donation be to motivate you to participate in a network study??)

Enter your answer here:

**[]**

**There are several ways in which you can be invited to participate in a network study. What do you think is the best method to get the invitation? In the right box, put the items in order from most to least desired. Put the most important item at the top.**

Your answers must be different.

Give a number for each option according to your preference from 1 to 4

| | |
|---|---|
| | a personal invitation from a friend via email |
| | an anonymous email invitation from the researcher |
| | a personal invitation from a friend via SMS or Whatsapp |
| | a personal invitation from a friend via Facebook |

## Recruit

The following questions are about factors that are important to encourage participants to invite their contacts to a network study.

For more information, click on the video:

**[]**

**There are several ways to invite someone to participate in a network study. What do you think is the best method of sending the invitation to someone? In the right box, put the items in order from most to least desired. Put the most important item at the top.**

Your answers must be different.

Give a number for each option according to your preference from 1 to 4

- [ ] I send the invitation personally by email
- [ ] I give the email address of my contact to the researcher so that an anonymous invitation can be sent
- [ ] I send the invitation personally via SMS or Whatsapp
- [ ] I personally send the invitation via Facebook

**[]**

**What type of compensation scheme do you prefer?**

Choose a maximum of one answer

Select all options:

- [ ] For every person you invite (max. 3 people) you will receive a 5 euro reward
- [ ] You receive 10 euros when you invite at least 1 person

[]

**What is important for you to decide to forward the invitation to participate in the network study to your social contacts?**

Choose the appropriate answer for each part:

| | absolutely not important | not really important | somewhat important | important | very important |
|---|---|---|---|---|---|
| The questionnaire does not take too long | ○ | ○ | ○ | ○ | ○ |
| The questions are not too Intrusive and personal | ○ | ○ | ○ | ○ | ○ |
| There is sufficient reward for completing the questionnaire | ○ | ○ | ○ | ○ | ○ |
| I think the study is important | ○ | ○ | ○ | ○ | ○ |
| Privacy of contact is protected | ○ | ○ | ○ | ○ | ○ |
| I get a good reward for sending the invitation | ○ | ○ | ○ | ○ | ○ |
| The questionnaire is easy and fun to complete | ○ | ○ | ○ | ○ | ○ |

In order for a network study to be successful, it is very important that participants invite their contact persons to also complete the questionnaire. How important are these factors to you?

[]

**What is the most important consideration for you when inviting contacts to do a health related study?**

Enter your answer here:

# RDSfeasibility

The following questions are about your social network.

In order for a network study to be successful, it is important that sufficient contacts can be invited to participate. Therefore, it is important to have an idea of how many social contacts you have and how many you can reach.

For more information, click on the video:

**[]How many gay men are in your social network that you communicate with via email, Facebook or text messages?**
*****

Choose one of the following options:

- 0-2
- 3-5
- 6-10
- more than 10

Please explain your answer here:

**[]Of all the gay men you know, with which part do you communicate via email, Facebook or SMS?**
*****

Choose one of the following options::

- with all of them
- with most of them
- with about half of them
- with less than half of them
- with none of them

Please explain your answer here:

# BeginNetworkStudy

Thank you for participating in this study.

**[]**

**Would you like to participate in a focus group discussion on the design of network studies?**

Choose one of the following options:

- ○ No
- ○ Yes

**[]**

**At which email address can we contact you?**

**Answer this question only if the following conditions are met:**
Answer was' Yes' to question '24 [FocusGroup] '(Would you like to participate in a focus group discussion about the design of network studies?)

Enter your answer here:

[ ]

The email address will only be used to invite you to this focus group.

**[]**

**We want to organize the focus group discussion in December or January.**

**On which days of the week are you (usually) available?**

**Answer this question only if the following conditions are met:**
Answer was 'Yes' to question '24 [FocusGroup] '(Would you like to participate in a focus group discussion about the design of network studies?)

Choose up to 3 answers

Select all options:

- ☐ Tuesday 18:00-19:00
- ☐ Wednesday 18:30-19:30
- ☐ Thursday 18:00-19:00
- ☐ none of these times

## []Is it okay if the focus group discussion is done in English?

**Answer this question only if the following conditions are met:**
Answer was 'Yes' to question '24 [FocusGroup] '(Would you like to participate in a focus group discussion about the design of network studies?)

Choose one of the following options:

- ◯ Yes, I agree
- ◯ No, I only participate if it is in Dutch

Please explain your answer here:

## []

## Previous network studies have shown that these studies are more likely to be successful if the first participants in a study are highly motivated.

## Would you like to participate in our network study and also invite your social contacts to participate?

Choose one of the following options:

- ◯ No
- ◯ Yes

## []At which email address can we contact you?

**Answer this question only if the following conditions are met:**
Answer was 'Yes' to question '28 [Seed] '(Previous network studies have shown that these studies are more successful if the first participants in a study are highly motivated. Would you like to participate in our network study and also invite your social contacts to participate?)

Enter your answer here:

The email address will only be used to invite you to the network study.

Submit your questionnaire
Thank you for participating in this questionnaire.
