# Supplementary material for: Improving web-based respondent-driven sampling performance among men who have sex with men in the Netherlands
Source: PLOS Digit Health. 2023 Feb 8;2(2):e0000192. doi: 10.1371/journal.pdig.0000192 (PMC9931300; doi:10.1371/journal.pdig.0000192)
Supplement: S1 Table — (PDF) [file pdig.0000192.s002.pdf]

Improving web-based Respondent-Driven Sampling performance among Men who have sex with men in the Netherlands

Supporting information

Table S1. Preferences regarding the DCE options ($N = 94$)

	**N (%)**
**Ideal Time of a study**	
<10 minutes	12 (12.8%)
10 - 20 minutes	54 (57.4%)
Approx. 30 min	16 (17.0%)
Approx. 45 min	3 (3.2%)
1 hour or longer	0 (0.0%)
Maximum time depends on something else	9 (9.6%)
**Preferred type of incentive**	
Donation	41 (43.6%)
Voucher	53 (56.4%)
**Preferred amount of incentive**	
Minimum €5	9 (9.6%)
Minimum €10	46 (48.9%)
Minimum €20	9 (9.6%)
More than €20	1 (1.1%)
I participate regardless of the reward	18 (19.1%)
Minimum would depend on something else	11 (11.7%)
