# Supplementary material for: Improving web-based respondent-driven sampling performance among men who have sex with men in the Netherlands
Source: PLOS Digit Health. 2023 Feb 8;2(2):e0000192. doi: 10.1371/journal.pdig.0000192 (PMC9931300; doi:10.1371/journal.pdig.0000192)
Supplement: S2 Table — (PDF) [file pdig.0000192.s003.pdf]

Improving web-based Respondent-Driven Sampling performance among Men who have sex with men in the Netherlands

Supporting information

Table S2. Analysis of the preferred Invitation/Recruitment Method by Age group

**Age category**	**Invitation Method**		**Recruiting Method**	
Variables	Coefficient	S.E.	Coefficient	S.E.
**18-34**				
Personal Email	0.856	0.450	0.205	0.432
Anonymous Email	-0.384	0.449	0.198	0.428
SMS/WhatsApp	0.789	0.455	1.035*	0.445
Facebook[a]	0	0	0	0
**35-44**				
Personal Email	0.867	0.468	1.291**	0.523
Anonymous Email	1.532**	0.489	1.170*	0.518
SMS/WhatsApp	1.059*	0.461	1.271**	0.523
Facebook[a]	0	0	0	0
**45+**				
Personal Email	1.876***	0.290	1.683***	0.300
Anonymous Email	0.753**	0.271	1.107***	0.286
SMS/WhatsApp	0.779**	0.261	1.186***	0.287
Facebook[a]	0	0	0	0

* $p < 0.05$; ** $p < 0.01$; *** $p < 0.001$

[a] Base alternative
